# Interactions between Invasive Monk Parakeets (*Myiopsitta monachus*) and Other Bird Species during Nesting Seasons in Santiago, Chile

**DOI:** 10.3390/ani9110923

**Published:** 2019-11-05

**Authors:** Cristóbal Briceño, Alejandra Sandoval-Rodríguez, Karina Yévenes, Matilde Larraechea, Angello Morgado, Catalina Chappuzeau, Víctor Muñoz, Pablo Dufflocq, Florencia Olivares

**Affiliations:** ConserLab, Animal Preventive Medicine Department, Faculty of Animal and Veterinary Sciences, University of Chile, Santiago 8820808, Chile

**Keywords:** affiliative interaction, agonistic interaction, Chile, conservation, ecosystem engineer, invasive species, monk parakeet, *Myiopsitta monachus*, nest occupancy, predation, review

## Abstract

**Simple Summary:**

The monk parakeet (*Myiopsitta monachus*) is an invasive species, unique in the parrot family for its ability to build large nest structures. This species became globally distributed, promoted in part due to the pet trade market, and now is considered a pest because of the economic losses they produce. During the reproductive seasons of 2017 and 2018, we registered interactions between invasive monk parakeets and resident bird species in Santiago, Chile. We observed positive and negative interactions, and herein, we describe parakeets’ nest occupancy by nine bird species, two invasive and seven native. For this reason, the monk parakeet should be considered an ecosystem engineer, a species that is creating available breeding space of potential use for other species. Our results contribute to an assessment of implications of this ecological invasion of local urban wildlife, and raise concern on other impacts, such as disease transmission, as a consequence of these interactions.

**Abstract:**

The monk parakeet (*Myiopsitta monachus*) is considered to be one of the most invasive bird species because its unique ability among parrots to build their own communal nests. Currently, they are considered an invasive species in 19 countries and a pest—even in their native distribution—because of economic losses derived from their impacts. During the reproductive seasons of 2017 and 2018, we registered interactions between invasive monk parakeets and resident bird species in Santiago, Chile. We observed agonistic and affiliative interactions, and further, we described monk parakeets’ nest occupancy by nine bird species, two invasive and seven native. For this reason, we consider that the monk parakeet is an allogenic ecosystem engineer with the potential to shape distribution and richness of sympatric species in urban environments. Our results contribute to an assessment of the implications of the monk parakeet’s ecological invasion to other synanthropic species, and raise concern of other potential impacts, such as pathogen transmission derived from these interactions.

## 1. Introduction

Invasive populations of monk parakeets (*Myiopsitta monachus*) can be found worldwide as an unintentional by-product of a large-scale pet trade [1]. The first published records of its invasion date from 1968 in Florida, USA [2], and they continue expanding their distribution through North America [3]. In Europe, the first description of population establishment was in Barcelona, Spain in 1975 [4]. Almost all research on the species has been conducted in Argentina in the parrot’s native range, and in USA and Spain on their invasive distribution. In Argentina, the monk parakeet is increasing its original range towards southern Patagonia [5]; in its native range, the species is currently considered a pest [3]. In England, where the monk parakeet is also increasing its population size, the species is classified as one of the six priority invaders for rapid control [6]. In the United States, parakeets build nests mainly on man-made structures, and they are considered a nuisance by utility companies [7,8,9]. In Barcelona, monk parakeet populations are estimated to double every nine years, a rate that is highly subsidized by bird feeders [10,11]. Both in their native and invasive distributions, they are considered to be a pest, primarily because of damages to agricultural crops, power structures, and the avian pet trade [5,12,13,14]. In Chile, there is little information about the invasion of monk parakeets, although it is considered to be the newest and most troublesome invasive bird species, producing major negative impact on fruit and ornamental trees [13,15]. This invasion has been completely overlooked, and it appears to be expanding to new urban, and even rural, areas [15,16]. It is not enough to extrapolate previous knowledge of the invasion of the species in other places; instead, it is critical to understand the factors favoring monk parakeets’ invasion at a regional-specific scale [17]. Furthermore, to prevent or decrease an alien species impact and/or further spread or damage, it is crucial to understand the ecology of the invasion, especially as dispersers colonize new areas [6,18]. On the other hand, lessons can be learned from local invasions to project impacts in other invasive distributions. In this case, potentially, parakeets’ interactions with other introduced birds such as sparrows (*Passer domesticus*) or rock pigeons (*Columba livia*) could pose a risk of transmission of pathogens, especially to immune susceptible individuals such as younger, older, or sick individuals [19,20].

Determining interactions established by invasive species with other animals is of great importance, since biological invasions can contribute to the extinctions of wild species and are tightly related to the emergence of diseases that may affect the health of human populations, domestic animals, and native fauna [21,22,23,24,25]. Reports around the world have described interactions established by monk parakeets and species from regions they have invaded. The parakeets have been described as being both hostile and tolerant towards other species of birds and mammals [12]. In the United States, when hungry, monk parakeets have been seen to aggressively intimidate other birds from approaching food and, sometimes, have caused the death of other avian species [26,27]. Parakeets aggressively defend their nests against other species [28]. Another introduced species, rock pigeons, have interacted with monk parakeets in Córdoba, Argentina, where they used parakeet’s nests until displaced by the parakeet, which blocked nest access with sticks [29]. Occupation of parakeets’ nests by native species, such as speckled teals (*Anas flavirostris*) and whistling ducks (*Dendrocygna* sp.) in parakeets’ native range in Eastern Argentina, has also been documented, with ducks being observed occupying abandoned nests [28,30]. The American kestrel (*Falco sparverius*) has also been reported to breed in parakeets’ nests in Argentina; most of the times abandoned, but also sharing the same large nest structure with the parakeets, although in a different chamber [31]. Other birds, such as the guira cuckoo (*Guira guira*), the grayish baywing (*Agelaioides badius*), the screaming cowbird (*Molothrus rufoaxillaris*), and the white monjita (*Xolmis irupero*), are known to occupy monk parakeet’s nests in Córdoba Argentina. Further, nest sharing between monk parakeets and the lark-like brushrunner (*Coryphistera alaudina*) has been noted in the Paraguayan Chaco, where these two species built nests collectively [32]. Likewise, apparent pacifist nest sharing between this psittacid and the tropical screech owl (*Otus choliba*) was described in Argentina [30]. Also, in their native range in Mostardas, Brazil, introduced house sparrows were reported deploying agonistic behavior towards monk parakeets, as a group of male sparrows expelled a group of parakeets from their nest and later occupied it [33]. Invasive monk parakeets have also been seen to kill introduced house sparrows in Pennsylvania, USA, while defending their territory [26]. In sum, there is a growing body of behavioral and ecological literature demonstrating a wide array of interactions between this invasive species and resident ones.

Scarce information on the ecology of the invasion of the monk parakeet has been produced in Chile, where this invasive species is expanding its distribution to new urban and rural areas [15,16]. Ecological interactions between monk parakeets and other bird species in Chile remain largely unknown. The only report of parakeet interaction from Chile describes native American kestrels preying upon monk parakeets in Santiago. Twice, adult kestrels preyed upon and obtained live monk parakeets’ chicks in their nests to feed themselves and their young on the same and neighbor trees. In both instances, adult parakeets fled the nest and vocalized from neighboring trees, but never defended their nests against the kestrels [34]. In addition to the American kestrel, other native bird species in sympatric urban parks in Santiago during the spring include the Austral thrush (*Turdus falcklandii*), eared dove (*Zenaida auriculata*), rufous-collared sparrow (*Zonotrichia capensis*), southern lapwing (*Vanellus chilensis*), yellow-winged blackbird (*Agelaius thilius*), Austral blackbird (*Curaeus curaeus*), house wren (*Troglodytes aedon chilensis*), blacked-chinned siskin (*Sporagra barbata*), white-crested elaenia (*Elaenia albiceps*), chimango caracara (*Milvago chimango*), tufted tit-tyrant (*Anairetes parulus*), rufous-tailed plantcutter (*Phytotoma rara*), common diuca-finch (*Diuca diuca*), plain-mantled tit-spintail (*Leptasthenura aegithaloides*), Chilean mockingbird (*Mimus thenca*), long-tailed meadowlark (*Sturnella loyca*), Chilean swallow (*Tachycineta meyeni*), and the grassland yellow-finch (*Sicalis luteola*) [35]. Besides the monk parakeet, introduced birds that may be found in Santiago during spring are the rock dove, house sparrow, and shiny cowbird (*Molothrus bonariensis*) [35,36].

The present work describes observed interactions between invasive monk parakeets and other bird species during two reproductive seasons (2017 and 2018) in Santiago, Chile. Our aim herein is to assess potential ecological interactions which may result in benefits or detriment for sympatric bird species. Further, given the unique characteristic of monk parakeets to build their own complex nests and the interactions registered, we propose that the monk parakeet should be considered an ecosystem engineer, and its ecological impacts should be assessed from broader perspectives that include native species, urban health, and associated economic costs.

## 2. Materials and Methods

### 2.1. Study Area

The study was conducted in Santiago (33°27’ S; 70°38’ W), the capital city of Chile located in the Metropolitan Region. The Region is located in the Mediterranean bioclimatic zone of Central Chile, characterized by dry summers and wet winters, with strong inter-annual variability due to El Niño-Southern Oscillation phenomenon [37]. Mean annual temperature is 13.2 °C, and the mean annual precipitation is 531 mm [38]. Temperature and moisten patterns are primarily a function of topography, resulting in a vegetation mosaic of *Acacia caven* shrubland at lower hillslopes, and evergreen sclerophyllous forest, mainly in drainage corridors and southern aspect slopes [38,39].

The Metropolitan Region has 52 communes and concentrates 40% of the national population with 7,112,808 inhabitants, making it the highest densely populated region of the country, with 462 people/km^2^ [40]. This area has experienced a profound landscape transformation since the mid-sixteenth century, mainly due to logging, urban and agriculture expansion, livestock overgrazing, and introduction of invasive species [38,41]. Further, Santiago city is considered to have quickly grown in a disorganized manner, fueled by neoliberal policies over the last decades, resulting in urban sprawl with intense environmental degradation [42]. Currently, 96.3% of the metropolitan human population lives in urban environments [40], in a region acknowledged as one of the world’s 25 biodiversity hotspots [43].

### 2.2. Study Species

Monk parakeets are medium-sized sexually monomorphic parrots native to Paraguay, Uruguay, Bolivia, southern Brazil, and northern and central Argentina [2,44]. Subsidized by the pet market, monk parakeets were introduced in many areas and are currently classified as invasive in 19 countries, including several European territories, North America, Asia, and Chile, among others [1,3,15,45]. Their widespread success is attributed to behavioral and ecological traits, such as their flexible diet, gregarious behavior, tolerance to human disturbance and novel habitats, and high population growth rates as a result of their reproductive strategies [1,5,12,46,47]. In fact, recent studies have recognized the higher invasive success of monk parakeets on their invasive range, compared to their native habitats [48].

Perhaps its key invasive feature is that the monk parakeet is the only species belonging to the order Psittaciformes that is able to build its own communal nest, making it independent of tree or cliff cavities [46]. The monk parakeet’s nest is a structure of sticks containing one or more chambers. A chamber is a cavity in a nest, which may be occupied by a monogamous couple, albeit all individuals of the colony, including juveniles, participate in nest construction and maintenance [49]. These nests may weigh up more than 1000 kg [10,50]. As non-migrants, they remain in their nests for nesting and roosting year-round [51]. It is estimated that in their native distribution, 30 to 60% of chambers will be occupied for breeding [44,52]. In Argentina, the pairs will clutch between three to seven eggs during late October to early November [44], with fledglings departing the nest at 40 days of age [53]. Although *M. monachus* is considered to display marked philopatry and have limited dispersal in their native range [46], genetic evidence has found long distance dispersal in introduced populations of the USA [54].

### 2.3. Study Methods

As part of a research project, monk parakeet nestlings were collected from nests throughout the city during 2017 and 2018. To identify sampling sites, vehicle access, and other logistic details, an extensive database of monk parakeets’ nests in Santiago was produced and associated parameters registered. These parameters included tree species and characteristics, number and size of nests, nest height, and nest occupancy. Most sites were visited more than once; at least a first visit once the nest was discovered and variables collected, and an additional visit during the reproductive seasons. During sampling visits, one or two researchers inspected nest chambers with an endoscope, and manually collected parakeets’ chicks with the aid of a 17 m person hydraulic lifting crane.

During these visits, we recorded data on relationships between monk parakeets and sympatric birds. These included the frequency of nest occupancy by other species, and other spatial information, including occupancy of the same tree, as well as diversity of flocks engaged in shared space. Observation units were the trees where monk parakeets nested. For the purpose of this study, we considered only observations that occurred between 15 August to 21 December of years 2017 and 2018. We did this because (1) this is the primary period of potential reproductive activity for the monk parakeet in Santiago, (2) we assume parakeets are more sensitive to the presence of sympatric birds, and (3) we encountered eggs and offspring during surveys. Finally, we chose a conservative wider window for reproductive period in order to avoid losing information, since in their native distribution, these periods vary between consecutive years, as well as the possibility of parakeets’ second clutches [16].

Interactions between monk parakeets and sympatric bird species were classified using a simple ethogram based on our observations [55,56,57]. Interactions were classified as affiliative when bird species (parakeet and other species) were “Foraging together” at the base of the tree or immediate surroundings. Agonistic interactions included “Alarm call”, when a parakeet produced repetitive vocalizations towards another bird species; “Attacks”, when a bird approached in straight flight and menacing; and, “Predation”, when another bird species was observed consuming a parakeet.

Additionally, all events of nest occupancy by other bird species were registered, either by direct visualization of eggs or offspring inside the monk parakeet’s chamber, or by direct observation of one or two conspecific (putative parents), repeatedly entering and leaving the chamber, as well as guarding the chamber entrance. Finally, although not considered as true interactions, we also recorded the frequency that another bird species perched or used the immediate grounds within the tree where parakeets had an active nest.

Data collection and nest sampling were coordinated with municipalities for logistics and authorization to conduct our research in public urban areas. Parakeet sampling was authorized by Servicio Agrícola y Ganadero (SAG; Chilean Fish and Wildlife Service) under permit No. 716/2016. Bioethical approval (No. 19-2016) and Biosafety approval (No. 82) were issued by the Faculty of Animal and Veterinary Sciences, University of Chile.

## 3. Results

Overall, we identified 547 trees with one or more parakeet nests, and one nest constructed over a lamp post, in 24 Communes of Santiago, mainly in public areas, such as streets, squares, and parks. We registered 374 observations in 234 different trees with at least one monk parakeet nest between 3 May 2017 and 2 May 2019. For the period comprised between 15 August and 21 December (Austral spring), we recorded 179 observations collected from 130 trees, all of which were identified to species level. Thus, observations occurred in 25 tree species, listed in Table 1. From the 179 observations, 19 (10.6%) corresponded to true interactions represented by “Forage together” (*n* = 4), “Attack” (*n* = 14), and “Predation” (*n* = 1), while the remaining observations corresponded to “Occupy same tree” with 138 observations (77%) and “Occupy nest” with 22 (12.3%). More than half of the observations of parakeets occupying the same tree with other species occurred with rock doves (21.7%), Austral thrushes (17.4%), chimango caracaras (13%), and Austral blackbirds (10.1%; Table 2).

Agonistic interactions between sympatric bird species and monk parakeets were observed occurring on 15 occasions with three species: Chimango caracara (80%), Harri’s hawk (*Parabuteo unicinctus*; 13.3%), and Austral blackbird (6.7%). From these interactions, only one event corresponded to “Predation” of a parakeet by a Harri’s hawk. On the other hand, affiliative interactions occurred on four occasions, as “Forage together” was noted between parakeets and the eared doves (50%), rock doves (25%), and Austral thrushes (25%; Table 2).

**Figure 1:**
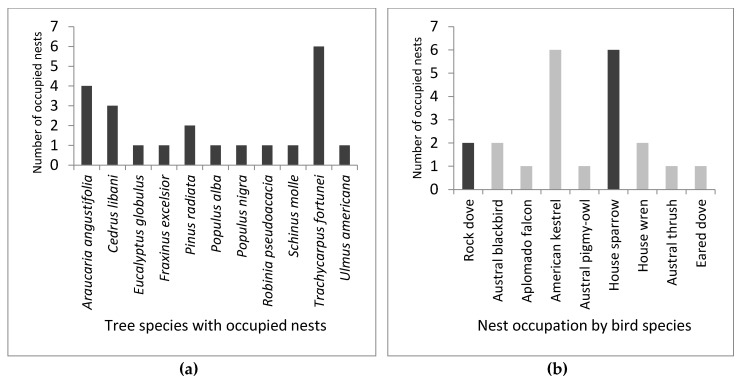
Monk parakeets’ nest occupation events (*n* = 22) by sympatric bird species during Austral nesting seasons of 2017 and 2018 in Santiago, Chile. The ordinate axis shows frequency of events, while the abscissa list species: (**a**) Tree species with occupied nests; (**b**) nest occupation by bird species. Darker columns inform introduced species, while lighter columns inform native species.

**Figure 2:**
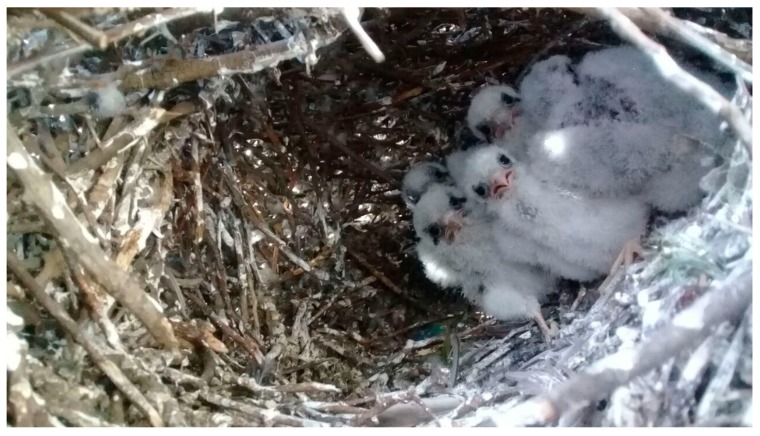
American kestrel (*Falco sparverius*) nestlings bred in a monk parakeet nest chamber, during the Austral spring of 2018 in Santiago, Chile.

We found 22 monk parakeets’ nest chambers being occupied by nine different bird species, each event found on a different tree unit. Hence, occupied chambers were found at least more than once on Chinese windmill palms (27.3%), Paraná pines (18.2%), cedars of Lebanon (13.6%), and Monterey pines (9.1%; Figure 1a). Nest occupation events were observed more frequently by American kestrels (Figure 2) and house sparrows with 27.3%, followed by rock doves, Austral blackbirds, and house wrens with 9.1% (Figure 1b).

## 4. Discussion

There is yet much to be learned from free-ranging parrot behavior, as social behavior of most psittacine species has been studied in captive populations, where birds are commonly housed in pairs [58]. This is particularly important with invasive species, as they are recognized to be one of the main threats to native biodiversity globally [59]. The monk parakeet is widely distributed through the city of Santiago, where little is known about the relationship that this invasive species may establish with other resident synanthropic birds. During the Austral nesting seasons of 2017 and 2018, we were able to opportunistically observe interactions, parakeets’ nest occupancy, and space share at tree level between the parakeets and sympatric bird species. We registered 179 observations of this kind in 130 trees of 25 species, each having at least one monk parakeet nest. From these species, 11 (44%) were deciduous and 14 (56%) perennial, while 23 (92%) were introduced and the remaining two (8%) were both native and endemic of Central Chile, a biodiversity hotspot with global conservation priority [43]. In regard to space share within trees, monk parakeets where observed sharing space with 17 sympatric bird species with no apparent distress (Table 2), perhaps related to the fact that monk parakeets do not defend an all-purpose territory [49]. More than half of these observations occurred with rock doves (21.7%), austral thrushes (17.4%), and chimango caracaras (13%). From all these species, rock doves, shiny cowbirds, and house sparrows were also introduced [36].

We narrowed our observations to reproductive seasons for two reasons: First, because of the effort. Although we recorded interactions throughout the year, given our study design, most of our fieldwork—and observations—were conducted during the Austral spring, when birds are actively deploying courtship and breeding. The second is that we would expect monk parakeets to be more alert towards other bird species, given their reproductive behavior, thus increasing their sensitivity to potential competitors and, therefore, detectability by observers. Although underlaying mechanisms behind bird behavior during reproductive seasons are not obvious [60], we would still expect that parental care and frequency of nest visits would be high, especially due the nature of monk parakeet offspring, which are altricial and, thus, highly dependent on parental care [53]. Consequently, it has been described that territorial defense in monk parakeets is restricted to nest colonies [49].

In regard to observed interactions, parakeets foraged alongside other bird species. Given that it has been described that larger monk parakeet flock groups provide more time to feed, as birds will invest less time in scanning for predators [61], we believe that this observation can be classified as an affiliative interspecific conduct between parakeets and introduced rock doves, or natives Austral thrush and eared dove. More interaction events were observed for agonistic conducts (15 versus 4 affiliative), where the majority of these observations occurred between monk parakeets and chimango caracaras (80%), followed by Harri’s hawks (13.3%) and Austral blackbirds (6.6%). From these observations, the only depredation was observed by a Harri’s hawk. The Austral blackbird was also seen occupying a parakeet’s nest. Interestingly enough, all agonistic interactions were observed with native bird species.

Perhaps the most characteristic feature of the monk parakeet is its unique ability—among psittacids—to build their nests with twigs and branches. These complex structures, which parakeets build communally, include several isolated chambers and may weigh hundreds of kilograms [10,50,62]. The nests are occupied throughout the year and may be used by several members of a colony, as reproductive couples, non-reproductive helpers, or parakeets in developing stages [46,49,50,51]. The nest also provides protection for offspring against predators and microclimate control [63]. Further, the nests that monk parakeets construct have been described to represent complex structures with thermoregulatory buffer capacity, which is important in hot and cold climates, increasing parakeets’ invasive potential [64]. Both in their native and introduced distributions, parakeets’ nests have been observed to be colonized by other bird species [29,30,31,32,33]. We were able to confirm parakeets’ nest use by nine resident species in 22 occasions in Santiago. From these, only the rock dove (9.1%) and house sparrow (27.3%) were introduced, while the rest were all native. Thus, the American kestrel (27.3%), Austral blackbird (9.1%), house wren (9.1%), aplomado falcon (4.5%), Austral pigmy-owl (4.5%), Austral thrush (4.5%), and eared dove (4.5%) are native species making use of an invasive species’ resource during their reproductive cycle. Monk parakeets’ nest occupation by the American kestrel has been described in Argentina [31]. The American kestrel, aplomado falcon, and Austral pigmy owl are birds of prey, which may have been attracted to the parakeet’s chambers in search of food. Nonetheless, we were unable confirm this, as we observed these raptors breeding in the nests, but not the interaction of the first occupation itself. Additionally, we observed a rock dove using a parakeet nest in July, during Austral winter, not for reproductive purposes, but likely for climate protection. We also observed a monk parakeet nest with four parakeet eggs, but a different spotted egg which may have been from the introduced shiny cowbird, which is known to perform brood parasitism [65].

Our observations of several species using parakeets’ chambers for reproduction are important, as the monk parakeet may be shaping resident bird distribution and richness, while creating a reproductive niche, through nest construction within the city. In Santiago, there is no other bird species that constructs such complex nest structures, and the monk parakeet is widely distributed in urban areas. Given reported observations of monk parakeets’ nest use by other bird species, plus our confirmation of this occurring with several species in Chile, we propose that the monk parakeet should be considered as an allogenic ecosystem engineer due its capacity to construct and confer a reproductive resource to other species [66,67]. This is relevant, as recognizing ecosystem engineers, especially in their invasive distribution, allows a better assessment of the influence these species may have upon resident species they share habitat with [68]. Further, invasive species that are able to modify the ecosystem through creation of physical resources, and thus, increase habitat complexity, are those that have the largest impact [69]. For instance, in Madrid, the stock dove (*Columba oenas*) seems to have increased its distributions and abundance in urban areas through the use of monk parakeets’ nests [70].

Monk parakeets’ nests may be a source of parasites or pathogens for sympatric bird species that use these structures [71]. In their native distribution, monk parakeets have been observed to have heavy parasite loads to which nestlings are particularly sensitive [49,72]. Some of these parasites have also been described for the first time in Chile, and associated with the introduced monk parakeet [15]. Monk parakeets are known to occupy fresh plant material that may be of use as antiseptics to reduce pathogen load in nests [64,73]. We have observed monk parakeets collecting fruits of the chinaberry tree (*Melia azedarach*) but not consuming it, and we have also found the fruit within their nests, likely related to high pathogen loads in closed chambers, as well as the insecticidal properties of this urban introduced tree in Santiago [64,74,75]. The potential role of monk parakeets in spreading pathogens, such as ectoparasites, is of particular conservation interest, as biological invasions have been linked to the emergence of diseases in native populations [25].

Interactions of monk parakeets with other native or introduced taxa, such as mammals or reptiles, are also possible. In Argentina, monk parakeet depredation by white-eared opossums (*Didelphis albiventris*) and Patagonian green racer (*Philodryas patagoniensis*) has been described [30]. In Santiago, urban park managers mentioned that black rats (*Rattus rattus*) were observed climbing tree trunks, entering monk parakeets nests, and depredating monk parakeets, something that has also been described in Barcelona, Spain [48]. Lastly, we have observed the use of thorny branches of the native Roman cassie (*Acacia caven*) in monk parakeets’ chamber entrances, likely as a defense barrier to these and other predators.

Even though our study is restricted to opportunistic observations during reproductive seasons, we believe that our methodology and conclusions allow to further understand the potential ecological implications of the invasive monk parakeet population in Chile. For the nature of the observed parakeet interactions, direct observation is an appropriate methodology, especially useful for short duration conducts [76]. Further studies of ecological and sanitary impacts of introduced monk parakeets upon native species are warranted.

## 5. Conclusions

The monk parakeet has become one of the most invasive bird species globally, which, along with the trade fueled by the pet market, has established in 20 new countries. Perhaps its key feature is the unique ability among the Psittacidae family to be able to build complex nest structures which may weigh hundreds of kilograms. We have described interactions between introduced parakeets and other species, and further, we have evidenced the presence of other bird species breeding in parakeets’ nests. Due this unique ability among parrots to create complex structures with twigs and branches, which allow them to create an urban niche in which other species may nest as well, we propose the monk parakeet as an ecosystem engineer. This definition has implications on how we understand the monk parakeet invasion and to prioritize actions to address this expanding population in Santiago.

## Figures and Tables

**Table 1:** Tree species having monk parakeets’ nests, and where observations of interactions with other bird species were registered during Austral springs (15 August–21 December) of 2017 and 2018.

Tree Species	Scientific Name	Frequency of Observation (%)
Paraná pine	*Araucaria angustifolia*	33 (25.4)
Cedar of Lebanon	*Cedrus libani*	18 (13.8)
Chinese windmill palm	*Trachycarpus fortunei*	18 (13.8)
Black locust	*Robinia pseudoacacia*	11 (8.5)
Tasmanian bluegum	*Eucalyptus globulus*	9 (6.9)
Monterey pine	*Pinus radiata*	6 (4.6)
American pepper	*Schinus molle*	6 (4.6)
Black poplar	*Populus nigra*	5 (3.8)
Common ash	*Fraxinus excelsior*	3 (2.3)
Canary palm	*Phoenix canariensis*	3 (2.3)
Bunya pine	*Araucaria bidwillii*	2 (1.5)
American elm	*Ulmus americana*	2 (1.5)
Desert fan palm	*Washingtonia filifera*	2 (1.5)
Box elder	*Acer negundo*	1 (0.8)
Norfolk Island pine	*Araucaria heterophyla*	1 (0.8)
Sweet chestnut	*Castanea sativa*	1 (0.8)
Atlas cedar	*Cedrus atlantica*	1 (0.8)
Chilean acorn	*Cryptocarya alba*	1 (0.8)
Monterey cypress	*Cupressus macrocarpa*	1 (0.8)
Ceibo	*Erythrina umbrosa*	1 (0.8)
Chilean wine palm	*Jubea chilensis*	1 (0.8)
Sweet gum	*Liquidambar styraciflua*	1 (0.8)
White poplar	*Populus alba*	1 (0.8)
Water oak	*Quercus nigra*	1 (0.8)
Weeping willow	*Salix babylonica*	1 (0.8)
Total		130 (100)

**Table 2:** Observations of introduced monk parakeets surrounding their nest and resident bird species, between 15 August and 21 December of 2017 and 2018 in Santiago, Chile. Latin name of tree species are given in the Table 1.

Bird Species	Tree Species	Observation (No. Records)
Tufted tit-tyrant (*Anairetes parulus*)	Black locust	Occupying same tree (1)
Rock dove (*Columba livia*) ^1^	Box elder; Paraná pine; Bunya pine; Norfolk Island pine; Sweet chestnut; Cedar of Lebanon ^2^; Monterey cypress; Tasmanian bluegum; Canary palm; Black locust; American pepper; Chinese windmill palm ^2^; American elm; Desert fan palm	Occupying same tree (30) Occupying nest (2) Forage together (1)
Austral blackbird (*Curaeus curaeus*)	Paraná pine ^2^; Cedar of Lebanon; Ceibo; Black poplar; Chinese windmill palm	Occupying same tree (14) Occupying nest (2) Attacks (1)
Common diuca finch (*Diuca diuca*)	Paraná pine	Occupying same tree (1)
Aplomado falcon *(Falco femoralis*)	Tasmanian bluegum ^2^	Occupying nest (1)
American kestrel (*Falco sparverius*)	Paraná pine ^2^; Monterey pine ^2^; White poplar ^2^; Black locust; American pepper ^2^; American elm ^2^	Occupying same tree (7) Occupying nest (6)
Austral pigmy-owl (*Glaucidum nanun*)	Common ash ^2^	Occupying nest (1)
Chimango caracara (*Milvago chimango*)	Box elder; Paraná pine; Bunya pine; Cedar of Lebanon; Tasmanian bluegum; Chilean wine pine; Monterey pine; White poplar; Black locust; Weeping willow; American pepper; American elm	Occupying same tree (18) Attacks (12)
Chilean mockingbird (*Mimus thenca*)	Cedar of Lebanon; Common ash; Black poplar; American pepper	Occupying same tree (4)
Shiny cowbird (*Molothrous bonariensis*) ^1^	Cedar of Lebanon; Tasmanian bluegum; Chilean wine palm; Monterey pine	Occupying same tree (5)
Harri’s hawk (*Parabuteo unicinctus*)	Water oak	Attacks (1) Predates (1)
House sparrow (*Passer domesticus*) ^1^	Paraná pine; Common ash; Sweet gum; Canary palm; Monterey pine ^2^; Black poplar ^2^; Chinese windmill palm ^2^	Occupying same tree (11) Occupying nest (6)
Rufous-tailed plantcutter (*Phytotoma rara*)	Ceibo	Occupying same tree (1)
Green-backed firecrown (*Sephanoides sephaniodes*)	Black locust	Occupying same tree (2)
Chilean swallow (*Tachycineta leucopyga*)	Chilean wine palm	Occupying same tree (1)
House wren (*Troglodytes aedon chilensis*)	Cedar of Lebanon ^2^; Black locust ^2^; Chinese windmill palm	Occupying same tree (4) Occupying nest (2)
Austral thrush (*Turdus falcklandii*)	Paraná pine; Norfolk Island pine; Sweet chestnut; Atlas cedar; Cedar of Lebanon; Chilean acorn; Canary palm; Black locust; Chinese windmill palm ^2^; American elm	Occupying same tree (24) Occupying nest (1) Forage together (1)
Striped woodpecker (*Veniliornis lignarius*)	Monterey pine	Occupying same tree (2)
Eared dove (*Zenaida auriculata*)	Paraná pine; Cedar of Lebanon ^2^; Chilean acorn; Chilean wine palm; Black poplar; Black locust; American pepper; Chinese windmill palm	Occupying same tree (11) Occupying nest (1) Forage together (2)
Rufous-collared sparrow (*Zonotrichia capensis chilensis*)	Sweet gum; Desert fan palm	Occupying same tree (2)

^1^ Invasive bird species in Chile. ^2^ Tree species having parakeets’ nests occupied by the listed sympatric bird species.
